# 3D Printed Integrated Sensors: From Fabrication to Applications—A Review

**DOI:** 10.3390/nano13243148

**Published:** 2023-12-15

**Authors:** Md Sahid Hassan, Saqlain Zaman, Joshua Z. R. Dantzler, Diana Hazel Leyva, Md Shahjahan Mahmud, Jean Montes Ramirez, Sofia Gabriela Gomez, Yirong Lin

**Affiliations:** 1 Department of Aerospace and Mechanical Engineering, The University of Texas at El Paso, El Paso, TX 79968, USA; szaman3@miners.utep.edu (S.Z.); jzdantzler@miners.utep.edu (J.Z.R.D.); dhleyva@miners.utep.edu (D.H.L.); mmahmud4@miners.utep.edu (M.S.M.); jemontesramirez@miners.utep.edu (J.M.R.); sggomez@miners.utep.edu (S.G.G.); 2 Aerospace Center, The University of Texas at El Paso, El Paso, TX 79968, USA

**Keywords:** 3D printing, embedded sensor, additive manufacturing, sensor integration

## Abstract

The integration of 3D printed sensors into hosting structures has become a growing area of research due to simplified assembly procedures, reduced system complexity, and lower fabrication cost. Embedding 3D printed sensors into structures or bonding the sensors on surfaces are the two techniques for the integration of sensors. This review extensively discusses the fabrication of sensors through different additive manufacturing techniques. Various additive manufacturing techniques dedicated to manufacture sensors as well as their integration techniques during the manufacturing process will be discussed. This review will also discuss the basic sensing mechanisms of integrated sensors and their applications. It has been proven that integrating 3D printed sensors into infrastructures can open new possibilities for research and development in additive manufacturing and sensor materials for smart goods and the Internet of Things.

## 1. Introduction

Integrated sensors are microelectronic systems incorporated in a host material or structure and able to sense their exposed stimuli to produce an electrical output. Integrated sensors have been used in biology [1,2], energy [3], civil and mechanical structures [4], aerospace [5], and additive manufacturing [6] applications. Temperature, pressure, humidity, and motion are among the physical properties that can be detected by integrated sensors. Wang et al. sought to integrate the technology of structural health monitoring diagnostics for microelectronic systems [1]. Preventative measures were taken to reduce the risk of sensor failure and damage when integrated into the composite system. Various integration methods were tested, and low-cost pressure sensors were manufactured in this work. Petrie et al. investigated the effects of inserting sensors in silicon carbide (SiC) ceramics for monitoring the nuclear energy production process [3]. Sensor embedment was done by infiltrating cavities within SiC structures for nuclear reactor system monitoring. Parameters such as strain and fuel temperature were monitored for encapsulated material integrity and power operation productivity. 

Classifications of integrated sensors are based on their specific functions and implementation of the structure in the field of application. The types of integrated sensors that will be studied in this work are embedded or surface-bonded sensors. Embedded sensors are a network of technology that are directly incorporated into a material and can be integrated though direct embedment or by inserting into voids within the host material [7]. Shifts in stress concentration, crack development, and increased matrix stiffness are some issues that can be encountered when embedding sensors. Nevertheless, since the sensors are shielded from the outside environment, which reduces the risk of sensor damage and enhances durability. Surface bonded sensors are attached to the host structure surface using an adhesive [8]. Careful surface preparation must be done to effectively secure the sensor, and the bonding layer should be scaled accordingly. Sensing performance and the transducer ability to produce a signal through the bonding layer can be a setback for surface-bonded sensors. However, practical access to sensors suggests feasible sensor maintenance when experiencing failure.

Additive manufacturing (AM), also referred to as 3D printing or rapid prototyping, is the process where the material is deposited or joined in a layer-by-layer fashion to produce a three-dimensional part or object based on a digital model [9]. This type of technology has rapidly grown in popularity throughout the years due to its many benefits over conventional manufacturing methods. In comparison to traditional techniques such as computer numerical control (CNC) machining, injection molding, plastic forming, and plastic joining, AM technology has many advantages. These benefits include but are not limited to manufacturing cost, speed, part quality, and reliability [9,10,11]. AM costs are much lower than conventional technology in small volume manufacturing which requires expensive investments in mold development. It ensures fast prototyping and manufacturing, reduced time to market, and efficiency. This technique ensures innovation for customization, personalization, and the use of design imagination. AM technology keeps innovating and changing to increase its advantages and benefits over other manufacturing technologies [12,13,14,15]. 

The essential part of embedded/integrated sensing is that it cannot function without proper connections of functional materials (sensing part) with electrically conductive materials (communication part). In traditional manufacturing methods, multiple steps are required to complete the production of a single sensor and integrate it into the structure. Compared to traditional methods, AM technology is highly advantageous because with multi-material printing, a fully functional sensor can be fabricated within a single step in multi material printing [16]. The degree of freedom available when designing a sensor is incomparable to any other conventional technology [17]. Because of the unique set of advantages of AM methods, instead of competing with other traditional methods (computer numerical control (CNC) machines, hot pressing, and molding approaches), it is more likely that AM will complement other fabrication methods. Currently, there are different AM methods to combine functional material with conductive parts to enable sensing functionality. Hybrid AM method combines AM-printed parts with non-AM structures such as regular wiring, printed circuit boards, or entire sensors [18]. This method allows for specific combination of parts and complements other classic assembly techniques. Another method is conductor infusion that can print channels in otherwise non-conductive sensing materials by AM methods with a subsequent infusion of conductive inks [19,20,21,22]. In this method, the infusion of conductive materials in dielectric materials is possible by using dissolvable support material to form networks of channels. This method allows complicated electrical wiring to be printed since the channels are formed in full freeform fabrication [17]. The most complex and advantageous method to integrate sensors is multi-material printing that combines conductive and non-conductive materials [16,23]. Freedom of design, straightforward fabrication, and co-printing conductors, i.e., conductive materials printed in the same cycles as the dielectric materials, are the most desirable and positive sides of AM technology [17].

Many types of integrated sensors have been fabricated through AM that can sense, transmit, control, and react to environmental situations [24,25]. Embedded sensors have been explored through different AM technologies like fused filament fabrication (FFF), laser cladding (LC), stereolithography (SLA), ultrasonic AM, and laser powder bed fusion (LPBF). Fused filament fabrication (FFF) is the most used AM technology where the material is extruded through a nozzle and deposited layer by layer until a final part is fabricated. FFF is popular due to its simplicity, low cost, non-toxic, and cost-effective materials manufacturing process. Embedding sensors with FFF is possible by stopping the printing process and inserting the sensor into the enclosure. Sbriglia et al. embedded a single axis circuit piezoelectric accelerometers by stopping the printing process and inserting the sensor manually [26]. The application of FFF embedded sensors technology enables state-of-health monitoring and real-time diagnostics. Optimal depth placement of the sensor is important to get optimum sensitivity and accurate readings. Shemelya et al. created three varieties of capacity sensors using fine-pitch copper mesh and embedded copper wires by embedding an Aerotech gantry system [27]. A registration procedure was developed to record points in which the fused deposition modeling (FDM) machine would stop building the part to integrate the sensor and, subsequently, resume its fabrication until being fully embedded in polycarbonate material. The embedded bulk conductive sensors within the part contained wire, mesh, microcontrollers, and light-emitting diodes. These capacitive sensors successfully identified three metallic materials and saltwater from distilled water by measuring the relative capacitance when placed at the optimal depth. Embedded bulk conductive sensors have potential applications in biomedical, material sensing, electronic characterization, and electrical interconnect characterization. Stereolithography (SLA) AM technology uses directed irradiation to cure the light-activated resin in a vat layer by layer until a final part is fabricated [28]. Embedded sensors in SLA are seen more towards fabricating embedded micro-electromechanical system (MEMS) devices and electrochemical microfluidic devices (EMD). Tse et al. demonstrated that SLA could be used to create custom reaction packages of high aspect ratio to construct packages right on top of MEMS devices on a wafer scale. In addition, it provides the benefit of eliminating the dead volume of microfluidics and microsensors seen in traditional manufacturing [29]. Costa et al. fabricated a microfluid device with embedded low-cost reusable electrodes using stereolithography (SLA) [30]. EMD demonstrated good electrochemical stability, electroanalytical performance, and outstanding conductive performance. The SLA-printed EMD showed a suitable alternative tool for coupling separation techniques. Direct Energy Deposition (DED) is an AM technology that feeds a stream of metallic powder or wire into a melt pool that is created by a laser beam that scans across the coated targeted surface. Inkjet printing grants unprecedented deposition control up to the micron, resulting in accurate parts [31]. Due to the extreme dimensional accuracy of inkjet printing, it can produce fine seed circuits for small electronics. Ruikuan et al. utilized inkjet printing to fabricate a sensor system to monitor thermal flow, resulting in an energy efficient sensor with a linear performance [32]. Humber et al. similarly used inkjet printing, but for CO_2_ detection [33]. As seen with Ruikuan et al., inkjet integrated sensors are power efficient, with short carbon detection times and low power consumption. Hybrid AM incorporating traditional subtractive processing with AM, which enables the embedding and reconditioning of sensors using laser cladding. Juhasz et al. successfully designed a hybrid system using laser cladding to integrate a prefabricated functional ceramic ink-based strain gauge into a laser clad PH13/8 stainless steel enclosure [34]. Machining and laser cladding technology allowed for reconditioning of the sensor in case of damage. UAM enabled metallurgical bonds between layers of metal foils by using ultrasonic energy at room temperature. The low-temperature aspect of the process is attractive for embedded sensors as it can secure a safe integration of a sensor into a part without damage during the fabrication period. Hehr et al. embedded a fiber optic strain sensor into aluminum alloy 6061 ASTM tensile samples [35]. The embedded sensor showed no slipping, interface robustness, and accurate results during testing, with some limitations related to the commercially available fiber optic strain sensors being unable to read specific interface strain caused by poisons ratio effects. Laser powder bed fusion (LPBF) is one of the most popular AM technologies that involves using a laser on a powder bed to melt and fuse material powder [36,37]. On the topic of embedded sensing, LPBF faces a couple of challenges to make sure that the sensor is safe during enclosure (i.e., high temperatures, high pressures, powder contamination, chamber dimensions, inert gas flows, and the powder recoater). Binder et al., introduced design concepts to standardize LPBF embedding sensors [38]. The author embedded a pt100 temperature sensor which was previously embedded at the bottom of an elastic casting compound, to protect the sensor from electrostatics, and powder contamination, while still ensuring accurate measurements. An insulative aluminum cover was fixed to protect the sensor from direct laser radiation. Finally, all sensors were functional after embedding, but due to the embedding’s isolative effect, the sensor suffered from a delayed response.

**Figure 1:**
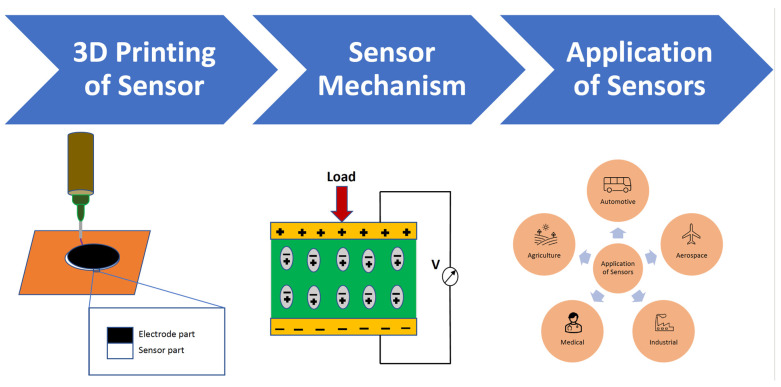
Basic sensor fabrication technique, sensing mechanism and applications.

This review article broadly discusses several types of integrated sensors with their sensing mechanisms, fabrication procedures, and embedding techniques using various AM processes. The basic sensor fabrication technique, sensing mechanism and applications are shown in Figure 1. It provides technical discussions of various integration methods for sensors with each AM technology. The most commonly and widely used techniques like FDM, Vat polymerization, DIW, DED, LPBF, and SLM are extensively described with their fabrication and embedding challenges, limitations, and recent progress of the works. The most distinguishable features of these embedded 3D printing technologies over conventional methods are their freedom in designing and ease of fabrication. This review also enlists some of the typical sensors explaining their construction methods using 3D printing, working principles and wide applications in different sectors. Lastly, the current limitations in embedding the sensors and 3D printing processes are discussed and future trends are also suggested to overcome existing problems. The 3D-printed integrated sensors, their fabrication methods, sensing mechanisms, and applications are summarized in Table 1.

## 2. Sensing Mechanism and Type

### 2.1. Transducing

Sensors are made up of the sensing component, a transducing mechanism, and an apparatus to interpret output data [39]. There are various types of sensing mechanisms based on physical or chemical principles. To distinguish which sensing element is suitable for a specific application, the characteristics of various transduction methods are discussed in the following section. 

#### 2.1.1. Piezoresistivity

**Figure 2:**
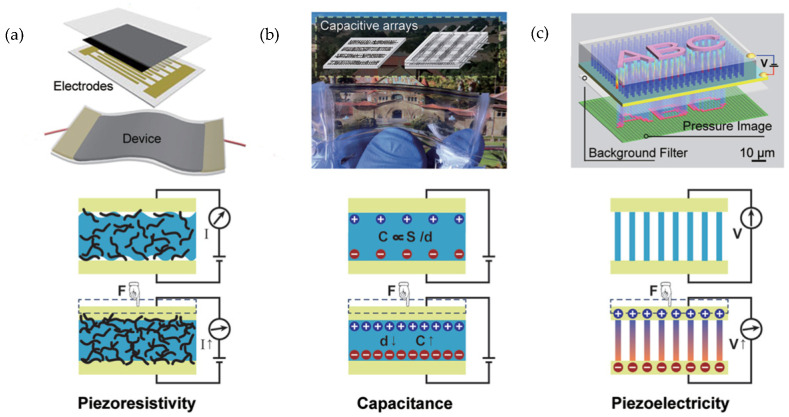
Schematic illustration of three common transduction methods and representative devices: (**a**) piezoresistivity (**b**) capacitance and (**c**) piezoelectricity [64]. Copyright 2015, Wiley Online Library.

Piezoresistive devices interpret variations of electrical resistivity within electromechanical systems while they are subjected to mechanical strain [40]. Piezoresistive mechanisms incorporate electrodes that can be embedded or attached to the device, as shown in Figure 2a. The structural mechanical, and electrical behavior of sensor materials, those of which should be electrically conductive, directly affects the performance of the piezoresistive response because of possible discrepancies in signal strength and accurate sensor readings. Wang et al. tackles common piezoresistive obstacles, such as signal sensitivity, by successfully 3D printing stretchable and porous sensing elements [41]. The electrode printing ink was comprised of plastic urethane and silver flakes while the sensing layer employed conductive carbon black nanoparticles and sacrificial sodium chloride particles for porosity.

**Table 1:** Fabrication, mechanism, and applications of 3D printed integrated sensors.

Methods	Printed Materials	Mechanism	Applications	Ref.
FFF	Thermoplastic elastomer	Capacitive	Force sensor	[42]
	TPU/PLA/Carbon black	Capacitive, Resistive	Mechanical and tactile sensing	[17]
	Polyphenylsulfone/Polycarbonate	Capacitive	Biomedical sensing, human interface devices, material sensing	[43]
	PA12/Magnetic particle	Magnetic	Magnetic sensor application	[44]
DIW	Sensor: TPU/Carbon black, Electrode: TPU/Ag	Piezoresistive	Skin-attachable electronics, human–machine interfaces, and electronic skins	[41]
	Silver with sacrificial ink	Inductive/capacitive	Food deterioration	[45]
	Graphene/PDMS and PTFE/PDMS	Electrical resistive	Smart textile	[46]
	Urethane Triacrylate/Methacrylic acid	Inductive/capacitive	Neuro-robotics and neuro-prosthetics	[47]
	Clay slurry	Capacitive	Relative humidity sensing	[48]
LPBF	Type K thermocouple	Seebeck effect	Temperature sensing	[49]
	SS 316L powder (Conductive material)	Magnetic	Structural health monitoring	[50]
SLM	SUS 316L, Inconel 718C	Thermal	Self-cognitive ability of metals	[51]
SLA	PDMS	Electrochemical	Biologically active molecule sensing	[52]
	Optical fiber	Pulse-calling	Particle analysis	[53]
DLP	Elastomer	Piezoresistive	Tactile sensor	[52]
	SP-RF0900	Resistive	Robotic manipulation	[54]
	Resin	Capacitive	Particulate matter sensing	[55]
DED	Ti-6AL-4V	Magnetic	Eddy current test	[56]
	Stainless Steel/Zirconia	Resistive	Structural health monitoring	[57]
Inkjet	Tin oxide	Electrical resistive	Gas sensing	[34]
	ZnO	Resistive	Gas sensing	[58]
	Acrylic rubber	Resistive	Robotic gripper	[59]
FDM	TPU/graphite ink	Capacitive	Robotics	[60]
	PLA/wax filament	Nucleotide sequence	Dengue virus detection	[61]
	BTO/MWCNT/PVDF	Piezoelectric	Energy storage	[62]
	BTO/PVDF	Piezoelectric	Pressure sensing	[63]

#### 2.1.2. Capacitance

The capacitive sensor consists of two parallel electrode plates and a dielectric material sandwiched in between [42]. The distance between the capacitor plates is directly influenced by the exerted force on the sensor, and the capacitance can be measured by also considering the plates’ overlying area. Qiu et al. fabricated integrated sensing capacitors to fabricate tissues and organs for surgery preparation through 3D printing technique [2]. The capacitance capability exhibited by their 3D printed sensors was accomplished through printing with polyacrylamide hydrogels for the plates and a silicone elastomer as the dielectric material, where the elastomer experienced deformation when compressed. Due to deformation, the tactile sensor produced a capacitance change directly related to the applied pressure that simulated organ/tissue handling during surgical procedures.

#### 2.1.3. Piezoelectricity

The piezoelectric effect translates applied mechanical energy into a voltage or generation of electric current [65]. Piezoelectricity is amongst the most efficient transduction methods, in terms of output voltage and high sensitivity [66]. The piezoelectric transducer is comprised of two electrodes that contain a piezoelectric material sandwiched in between; piezoelectric materials can be Lead zirconate titanate (PZT), Barium Titanate (BT) or Polyvinylidene fluoride (PVDF). Cui et al. prepared PZT colloidal particles for implementation into photo-sensitive ink to produce 3D-printed complex architectures [67]. The usage of 3D-printing enabled the ability to print convoluted geometries while maintaining a strong piezoelectric efficiency and therefore, functionalization of an additive manufactured part. Complete manufacturing of the piezoelectric devices follows the order, 3D printing fabrication, electrode formation, and poling. 3D printing makes it possible to merge the first two steps and make the poling process easier [9]. Figure 2 is showing three common transduction methods. 

#### 2.1.4. Magnetic Sensing

Magnetic sensors detect the presence of a magnetic field and provide actionable data regarding an object’s positioning, speed, rotation, and direction of movement. 3D printing technology presents a promising manufacturing technique to fabricate functional magnetic sensor devices of complex geometries with multiple materials and scales [68]. Only a few pieces of research in his field are available till now [44,56]. Christian Huber and his group mixed permanent magnetic filaments with pure polyamide (PA12) filaments and 3D-printed polymer-bonded magnets with a variable magnetic compound fraction distribution to obtain a required external field of the manufactured magnets [44]. Credi et al. proposed two different techniques for 3D printing high-sensitivity magnetically responsive cantilever beams and verified their feasibility as magnetic sensors [69].

### 2.2. Wired

3D Printing sensor technology can be considered as (a) embedding an existing sensor into a printed structure or (b) printing the entire sensor [60]. Electronic functionality has been added to additively manufactured parts by embedding wiring, printed circuit boards, or entire sensors. Integrated wired sensors can be obtained by joining a non-conductive material with conductive inks through previously printed channels or using multi-material printing of conductive and non-conductive materials [17].

Embedded sensors can be easily fabricated by manufacturing the non-conductive part first and then adding the electronic component. Shemelya et al. successfully fabricated capacitive sensors using fused deposition modeling and embedded wiring and were able to manufacture a fully encapsulated sensor [27]. To achieve this, the AM process was interrupted various times to fully embed all electronic components. In order to 3D print a joint-angle sensor, the fabrication process had to be halted once the cavity for the wiring harness has been printed to add this mentioned component to the part before printing is resumed. However, since the printing process must be interrupted multiple times during sensor fabrication, the procedure has to be organized and registered to maintain accuracy during the prints.

Sensors can also be fabricated by fusing a conductive material through channels fabricated in a non-conductive printed part. This approach for embedded sensors is challenging to implement because the fusion of materials makes it challenging to insert and remove supports in small spaces. With this method, the inks used can (a) remain liquid after infusion, (b) be infused as a liquid and then solidified via curing or evaporation of solvents, or (c) be infused as a solid via a carrier that evaporates after the process [70]. Chizari et al. developed highly conductive CNT/PLA nanocomposites to fabricate liquid sensors via 3D printing [70]. Here, the material was extruded out of a nozzle, allowing for tunable scaffold thickness affecting the relative resistance change inversely. The evaporation of solvent during the printing process raised issues of deformation, leading to filament overlap, and hence, more sensitive sensors. Utilizing the freedom that AM offers, Chizari et al. increased the number of printed layers, resulting in lower sensitivity. Mu et al. embedded silver nanoparticle ink via direct ink write into another 3D printed part for the use of flex sensors, leading to 9% yield strain, and low resistance change after cyclic loading and unloading [71]. TGA/DSC was conducted to ensure that the volatile solvent had been removed completely. This method of embedding sensors born of ink solvent removal was successful due to its use of limited supports and verification method. Mu et al. fabricated a flexible sensor, fabricating a ring that varies resistance based on the bent position of the finger. 

Fusion of materials via multi-material printing to fabricate sensors has the design freedom and is a straight-forward fabrication. Sensors fabricated using this method are primarily manufactured using ink or paste-based 3D printing technology such as direct ink write (DIW). Nassar et al. demonstrated the feasibility of this method by 3D printing a silver palladium paste and Glassbend Flexi material to fabricate a bendable smart sensing structure [23]. In comparison to the previous techniques, multi-material printing allows for the sensor to be manufactured in one single print without the need of interrupting or pausing the fabrication at the mid-print stage.

The challenge with wired embedded sensors, for all these methods, is that the sensors do have to be connected via a physical wire to a power source and to the component that will be outing the data provided by the sensor to have a fully functional sensor. Therefore, a new technology has emerged, allowing for wireless sensors to be fabricated via AM technology.

### 2.3. Wireless

Embedded printed components serve as efficient wireless sensors for accurate sensing, computation, and communication. These sensors shine in their capacity to monitor a wide range of physical and environmental variables, including pressure, temperature, motion, and others [72]. Wu et al., fabricated a passive wireless inductor-capacitor (LC) tank sensor using inkjet AM technology to create the coils channel and pad structures, which were later filled with liquid metal paint to create electrically conductive structures. This wireless LC tank sensor was used to measure the shift in resonance frequency which showed difference of 4.3% when the milk was stored at room temperature for 36 h [45].

Farooqui et al. pioneered the creation of 3D-printed disposable wireless sensors that incorporate microelectronics for extensive environmental monitoring. As a proof of concept, they demonstrated wireless temperature, humidity, and H2S level sensing [73]. Additionally, researchers have explored 3D-printed wireless implantable sensors. Herbert et al. developed a wireless, stretchable implantable biosystem via 3D printing for real-time monitoring of cerebral aneurysm hemodynamics, achieving wireless monitoring up to 6 cm through biological tissue [74]. Kalhori et al. designed and 3D printed a compact LC location sensor with enhanced wireless detection capabilities, enabling readouts from a distance of 10 cm [75]. Parker et al. created a customizable wireless implantable neural probe using 3D printing technology [76]. Furthermore, there have been reports on 3D-printed soft capacitive strain sensors integrated with wireless vascular stents, providing a biocompatible, battery-free, and wireless monitoring system [77,78].

## 3. Progress on 3D Printed Integrated Sensor

### 3.1. FFF Based 3D Printed Embedded Sensors

**Figure 3:**
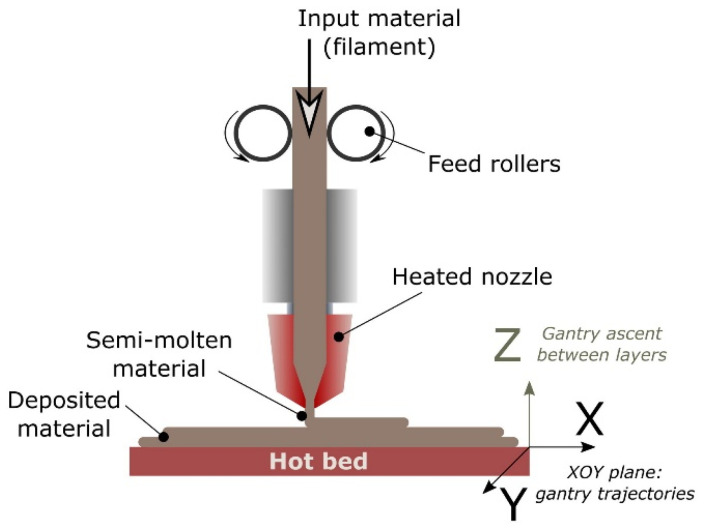
Process schematic of Fused Filament Fabrication (FFF) [80]. Copyright 2017, Elsevier.

**Figure 4:**
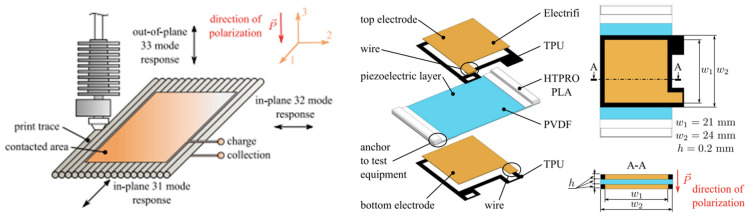
Coordinate system and mode response directions for a FFF piezoelectric PVDF film (**Left**), and single-process FFF dynamic sensor design with selected dimensions (**Right**) [80]. Copyright 2021, Elsevier.

Fused filament fabrication (FFF) is an AM technique where material is extruded through a nozzle and being deposited layer by layer until a final part is fabricated [79] shown in Figure 3. FFF technology has high potential in the creation of 3D printed parts with embedded sensors. Many works have demonstrated the capabilities of FFF technology to create a variety of sensors including electrochemical, capacitive, piezoresistive and piezoelectric. In piezoelectric sensors, Košir et al. designed a methodology for a single-process FFF manufactured dynamic piezoelectric sensor [80]. The piezoelectric sensor was manufactured by FFF made by polyvinylidene fluoride (PVDF) and poling with an electric field of 16.5 MV/m. Four different filaments were used for the 3D printed dynamic sensor: PVDF (piezoelectric film), electrify (electrodes), HTPRO PLA (build surface), and TPU (electrode support). Two types of sensors were manufactured using this technique to measure 31 (In-plane—direction along the print trace) and 32 (In-plane perpendicular to 31) piezoelectric mode responses as seen in Figure 4. Excitation force and the generated charge were the metrics used to measure the in-plane and out-of-plane piezoelectric responses. 

Katseli et al. fabricated an electrochemical cell-on-a-chip device by using FFF [81]. The device was manufactured in a single-step process using a dual extruder 3D printer. The printed part consisted of a miniature cell made with polylactic acid (PLA) with 3 electrodes embedded of carbon-loaded acrylonitrile butadiene styrene (ABS) conductive material as seen in Figure 4. The electrochemical sensor was used to determine paracetamol (PAR) and caffeine (CAF) in pharmaceutical tablets and was tested in urine spiked with PAR and CAR. 0.3 molL^−1^ of H_2_SO_4_ electrolyte was used due to having the best sensitivity and background characteristics for PAR and CAR. Differential pulse voltammetry (DPV) was used for simultaneous determination measurements of PAR and CAF. Lastly, the electrochemical chip design demonstrated fast and sensitive voltametric analysis while using small quantities of sample.

Gooding et al. manufactured a rectangular part with 3DSolutech Natural Clear PLA while embedding one layer into the surface, a 3D printed strain gauge using conductive PLA-Graphene composite filament [52]. The conductive material served to measure the strain gauge resistance by using the known geometry and bulk resistivity (0.6 Ω·cm) provided by the manufacturer. When connected to a circuit and providing supply voltage, (depending on the loading applied) the strain gauge will deform, and the resistance will change according to the geometry. When the gauge is strained, it will produce an output voltage variation that can be used to quantify the sensitivity of the embedded strain gauge. Three control factors were selected to examine the effects that geometry has on linearity, hysteresis, and repeatability to tensile load of the strain gauge specimen. These factors were the number of end loops, strand width, and thickness. In addition, due to the inconsistency of being able to print the complete strain gauge with one layer; different build orientations were explored to investigate the effects of conductive material through multiple layers. To validate the experimental data, finite elemental analysis (FEA) simulations were conducted. Results indicated that there was a difference between the FEA and the fabricated measured values due to inconsistent extrusion.

### 3.2. DLP/SLA Based 3D Printed Embedded Sensors

Photopolymerization-based 3D printing includes four types of technologies: stereolithography (SLA), digital light projection (DLP), PolyJet and two-photon polymerization (2PP) [82]. SLA uses a UV laser to cure resins layer by layer leading to 3D objects. DLP uses a projector (also referred to as digital light processing unit) that exposes UV light and cures a full layer of resin. PolyJet, developed by Stratasys Objet Geometries Co, Ltd., Rheinmünster, Germany, uses micronozzles that jet photopolymer resin droplets while simultaneously curing with UV light. 2PP is based on the simultaneous absorption of two photons in a photopolymer material.

**Figure 5:**
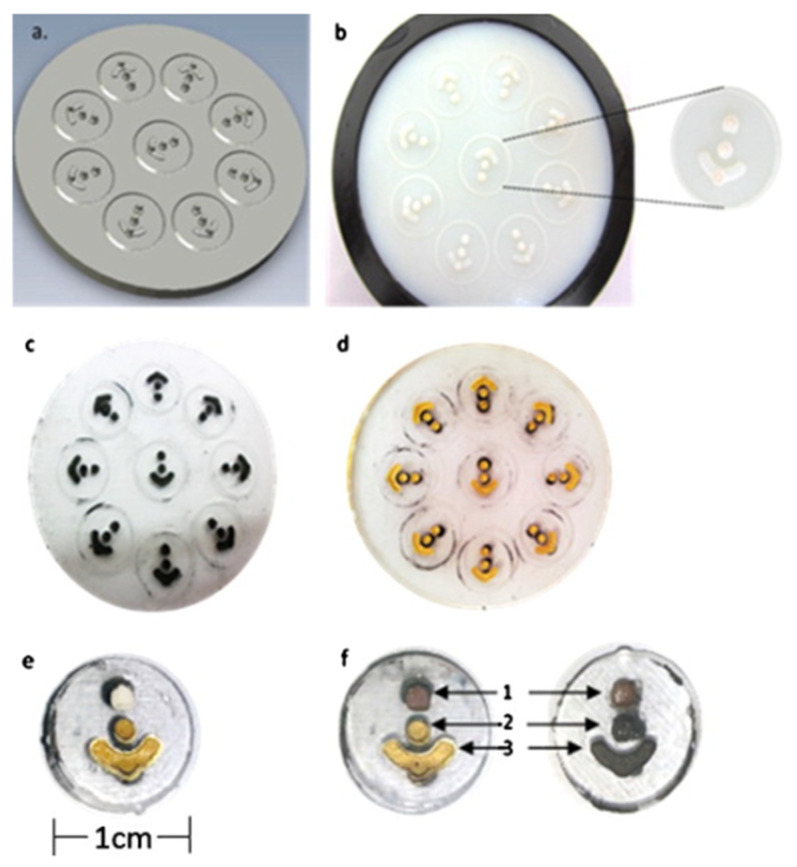
(**a**) Negative molds design; (**b**) 3D printed mold via SLA technology; (**c**) trenches and electrode patterns filled with PDMS conductive ink; (**d**) electrodes after Au sputtering; (**e**) electrodes after Au electroplating; (**f**) Au (**left**) and carbon (**right**) full chip [83]. Copyright 2015, Elsevier.

**Figure 6:**
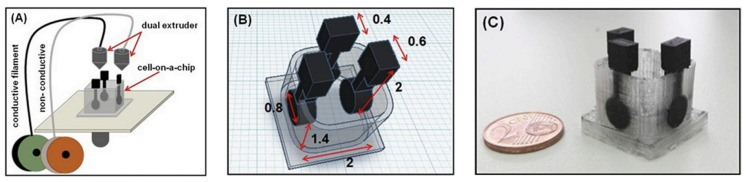
(**A**) Schematic presentation of the 3D-printing procedure for fabricating the cell-on-a-chip device using a dual extruder 3D printer. (**B**) The dimensions of the 3D-printed cell-on-a-chip device (in cm). (**C**) Photograph of the 3D-printed device [81]. Copyright 2019, Elsevier.

When it comes to embedded sensors, photopolymerization technology has a few limitations. Recently this AM process has facilitated the 3D printing of sensors. Initially, only single-material, rigid parts could be printed and photopolymerization primarily was used to fabricate molds in order to cast sensors. Ragones et al. fabricated a rigid mold via SLA which was then used to cast a PDMS chip that was used as the substrate for a biosensor [83] as shown in Figure 5. The trenches made on the casting based on the mold where then filled with conductive ink. Figure 6 details the fabrication process. In this study, a sensor capable of allowing a vertical detection approach on small volumes of cells and tissues without the need for transferring or removing the examined samples was successfully fabricated [2,83]. 

**Figure 7:**
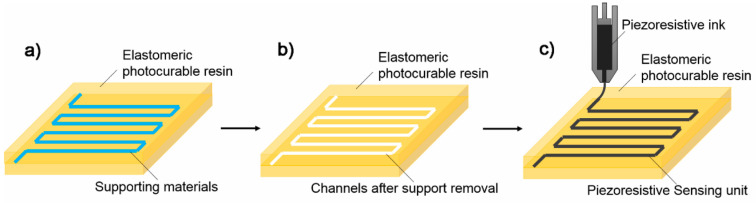
Indirect fabrication of sensors via printed channels: (**a**) sensor body with support material in channels space, (**b**) removal of supports to leave empty channels, (**c**) piezoresistive ink injections or direct ink writing [54]. Copyright 2018, MDPI.

The most common type of sensors fabricated via photopolymerization are those that function via previously printed channels, also known as hybrid 3D printing. Figure 6 depicts the fabrication of tactile sensors via DLP technology with the usage of conductive ink in printed channels [82] represented in Figure 7. 3D molds were printed through stereolithography to integrate flexible antenna- based pressure sensor with high sensitivity [54]. Hossain et al. also integrated flexible chip less RFID temperature memory sensor into 3D printed molds [84].

**Figure 8:**
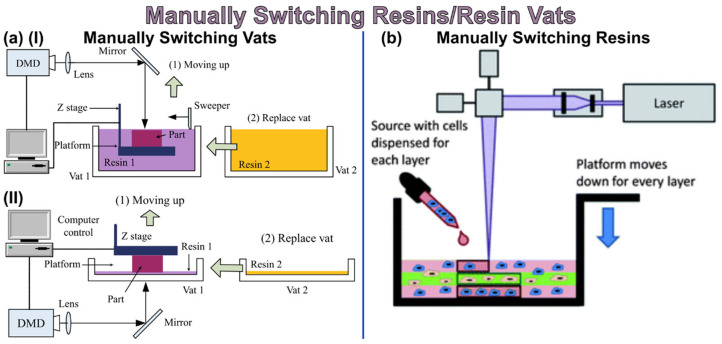
Examples of multi-material printing via Vat Photopolymerization technology: (**a**) manually switching vats(I) free surface and (II) constrained surface SLA system, (**b**) manually changing resins by injecting layer-by-layer [85]. Copyright 2021, American Chemical Society.

A more recent and growing DLP/SLA process for sensing is to manufacture sensors through multi-material printing. Before it was impossible due to the nature of this AM technology, however, in recent years new printers that have two vats and other ways to achieve this have matured. Some multi-material methods in Vat Photopolymerization include [85]:

(a) Manually stopping the print and changing vats/resins, as shown in Figure 8
(b) Injecting material for each layer
(c) Mechanical system changes vats/resins
(d) Printing material around a complex, preexisting 3D structure

**Figure 9:**
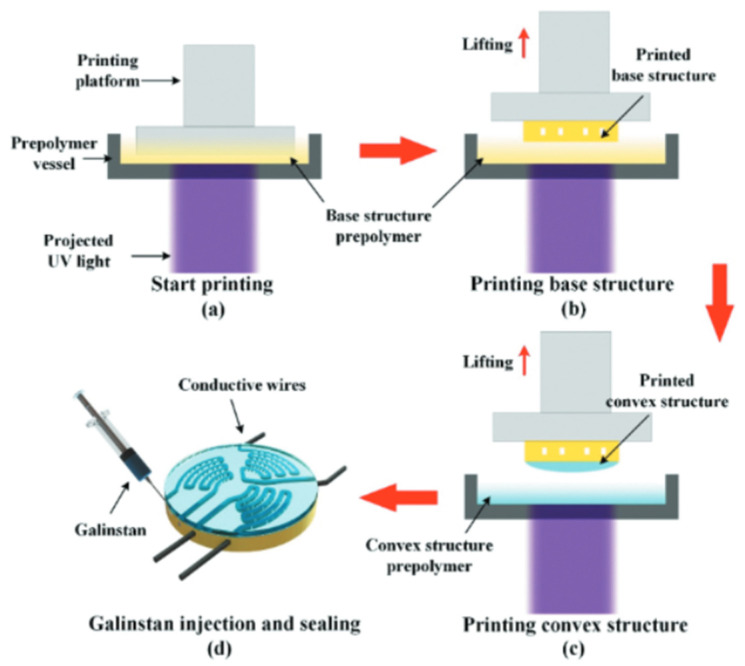
Step by Step fabrication process of tactile sensor by Wang et al. [86]. Copyright 2021, Taylor & Francis Online.

Wang et al. successfully 3D printed functional sensors with incorporated channels with DLP technology that were then injected with Galinstan metal [46]. The sensor structure was fabricated by multi-material printing of the sensor. First, the substrate with photosensitive resin was cured into the shape of a base structure with microfluidic channels. Then, the resin vat was changed to print a convex structure as the cover of the channels with a different photosensitive material. After the manufacture of the 3D printed part of the sensor, Galinstan liquid metal was injected into the microchannels as shown in Figure 9.

**Figure 10:**
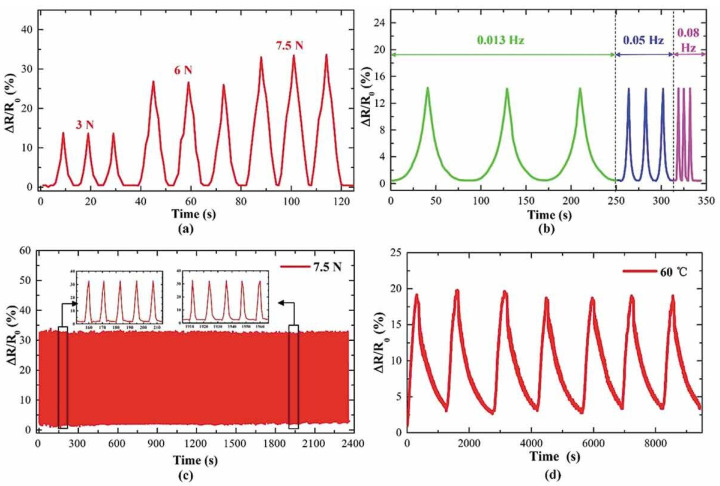
Sensing performance of tactile sensors: (**a**) resistance change under different loading forces, (**b**) resistance change under different loading frequencies, (**c**) 200 cyclic loading and unloading, (**d**) cyclic heating and cooling tests [46]. Copyright 2021, Taylor & Francis Online.

The tactile sensors were tested to sense different forces and temperatures. The applied forces increased consecutively between 0 N to 10 N under temperatures ranging from 20 °C to 60 °C [46] shown in Figure 10. The output voltages showed a linear increase for different temperature groups applied with the same forces. Furthermore, the resistance of the sensor increased as the temperature increased. An additional long-term multiple cyclic tests were conducted along with cyclic heating and cooling and cyclic loading and unloading tests. Wang et al., conducted 200 cycles that lasted 2400 s each with a loading force of 7.5 N and a frequency of 0.08 Hz.

### 3.3. Direct Ink Write Technique

Direct Ink Writing (DIW), also known as Robocasting, is a method associated with the material extrusion group. This method is generally used for non-Newtonian viscous slurry with composed rheological properties, as printing takes place at room temperature [86]. The DIW technique mainly contains two pieces of equipment, one is the software system that designs the structure, and the other is the output device that receives the motion instructions to complete the fabrication process. The dispenser or extruder moves according to the software, and materials are extruded through the nozzle generating the final part of the build platform. DIW has shown great potential for the development of 3D printed sensors with superior functional properties. The DIW method has some unique sets of advantages. For creation of embedded sensor technology, instead of competing with other traditional methods (casting, CNC machining, hot pressing, and molding approaches), this method complements them and can form a hybrid approach [16]. By using this process, the solid content in the final printed part can be higher compared with other AM processes [87]. Materials with properties similar to solid such as metals [88], ceramics [89] or wood [90] can be transformed into ink and printed. The number of research groups using DIW has expanded worldwide beyond the structural ceramics field into other areas, such as 3D bioprinting [91,92,93], energy [94,95,96], composites [97,98] sensors [22,99] robots [100]. Most sensors generally consist of multiple types of materials. Thus, a 3D printing method that can print different types of components, such as conductors [17], piezoelectric/dielectrics [16], flexible [101], and stiff materials [88], is key to the 3D printing of sensors [87]. DIW is perfect in this regard as with this technique, multiple types of materials can be printed in a single step with each having different parameters. There has already been a lot of research where DIW has been used to print embedded sensors. Vatani and his team adopted the method of DIW and were able to fabricate layered resistance sensors [102]. The team achieved the first 3D printed sensing arrays with CNTs inks where the 3D printed part was encapsulated in photocurable resin and PET to assemble the sensors. As a result, high-quality soft and flexible sensors with consistent sensing capabilities could be manufactured repeatedly with printable inks [49,103]. Kim and his team, directly printed a glove-type sensor that contained 10 strain gauges to measure flexion and extension of the five fingers, thus presenting a compact sensor system [99] and short production time. Shi and his team prepared aqueous ink mixing with polydimethylsiloxane (PDMS) sub microbeads/GO nanocomposite, which enabled high-resolution 3D DIW of strain sensors.

### 3.4. Laser Powder Based 3D Printed Embedded Sensors

Laser Powder Bed Fusion (LPBF) is an AM process that relies on fusing powders together through high energy lasers. The most common materials used are metals, like stainless steel, titanium, and Inconel, and polymers, like nylon [50]. There are a few different processes that have been researched to integrate sensors with LPBF. Oak Ridge National Laboratory reported efforts to embed thermocouples in stainless steel for the application of monitoring next-generation nuclear reactor temperatures [50]. Rather than embedding the sensors mid-print, channels on the build plate were machined using electrical discharge machining. Channel width and depth were varied to investigate the quality of the embedding process. Thermocouples were placed in these channels, spot welded, and sheathed using stainless steel (SS). After this, printing began to embed the thermocouples. To ascertain the sensor functionality, the thermocouples were exposed to thermal testing. This evaluation consisted of inserting the embedded sensors (along with a nonembedded control sensor) into a controlled furnace, and setting the temperature to 100 °C. The temperature increased by 100 °C increments to 500 °C, while holding the temperature for 1 h for each increment. The embedded sensors performed consistently to the nonembedded control sensor despite slight variation. At the beginning of the experiment, the embedded sensors recorded a lower temperature than the control. Later, the embedded sensors eventually recorded a higher temperature towards the end of the experiment when the control sensor began to match the embedded sensors. This discrepancy can be attributed to the time constant of heating the SS block that holds the sensors. The researchers predicted that, if all sensors were allowed to reach a steady state, the sensors would read the same temperature.

**Figure 11:**
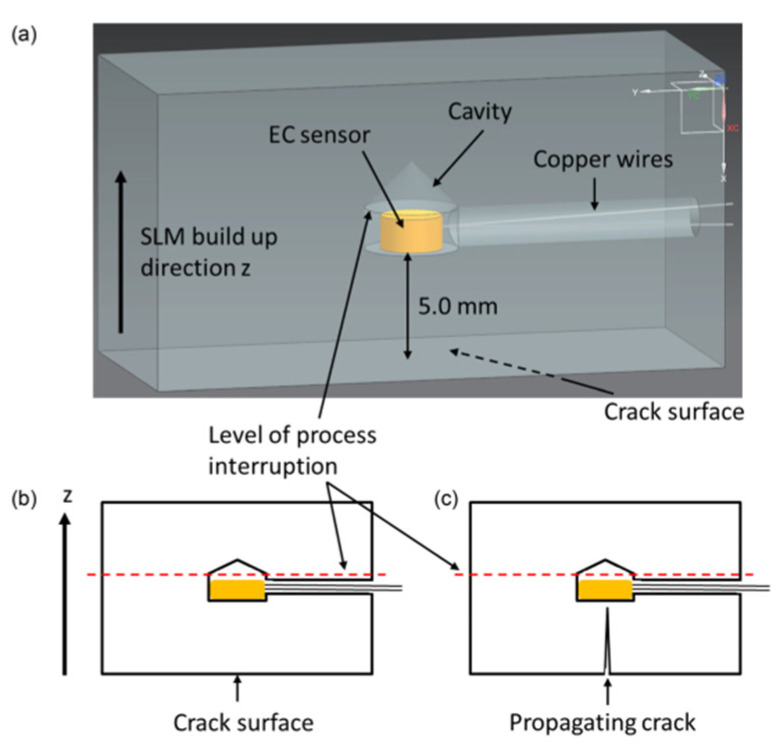
(**a**) CAD model of the demonstrator with dedicated cavity design for integration of EC sensors; (**b**) schematic representation of the demonstrator prior to crack initiation; (**c**) schematic representation of the demonstrator showing the crack propagating towards the embedded sensor [104]. Copyright 2021, Springer Link.

**Figure 12:**
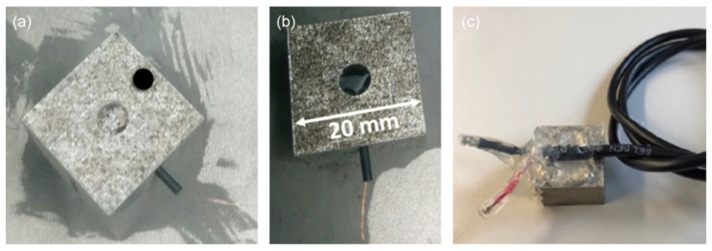
Sensor integration process for EC sensors: (**a**) powder removal, insertion of heat shrink tubes as wire protection and leading of wires through heat shrink tubes; (**b**) integration of the EC sensor into the cavity; (**c**) LPBF test specimens with soldered cables, ready to be tested [104]. Copyright 2021, Springer Link.

Embedded sensors introduce novel non-destructive testing methods. Stoll et al. integrated embedded eddy current (EC) sensors to enable structural health monitoring of SS 316 [104]. The study proves that embedded ECs can be utilized to observe crack propagation and determine damage severity over an extended period. SS 316 was chosen due to its low magnetic permeability, which coincides with the working principles of the EC, which utilizes magnetic fields for operation. Rather than embed the sensors during the AM process, a cavity was included in the CAD model, where the sensor was placed after selective laser melting (SLM). After pressing the sensor towards the bottom surface of the cavity, the entire cavity was filled with resin, as shown in Figure 11 and Figure 12. 

Fiber optic sensors have also been embedded using SLM technology. Havermann et al. manufactured SS 316 embedded sensors on a SS 316 substrate to determine strain levels, plastic deformation, and elastic deformation while using bare SS 316 samples to compare [51]. The embedding process includes a groove in the part, where the nickel coated Fiber Bragg Grating (FBG) sensors are placed. These sensors are covered by a layer of SS 316 powder, and are subsequently melted to the substrate, embedding the sample. Long-term elastic stability was investigated with this sensor. The sample was plastically deformed initially, but not in the later cycles. 

Placing the sensor in a pre-cut cavity and embedding during the printing process proves to be a popular method. H. Hyer and C. Petrie utilized SS 316 powders to embed a thermocouple and an optical sensor to measure strain. Embedding the sensors was a meticulous operation. Because the embedded thermocouple’s surface roughness and gaps needed to be minimized to reduce sensor response time, and the optical sensor requires near perfect embedding to sense strain [105]. With this, it also requires an embedded fiber and a floating fiber to separate the optical sensor’s ability to detect temperature and strain. Detecting the strain and temperature response is paramount in assessing the feasibility of integrated sensors. In the embedded region, the strain response is well observed. Adequate bonding is also documented, as the response is sensitive to strain while being independent of the temperature response during temperature testing. 

**Figure 13:**
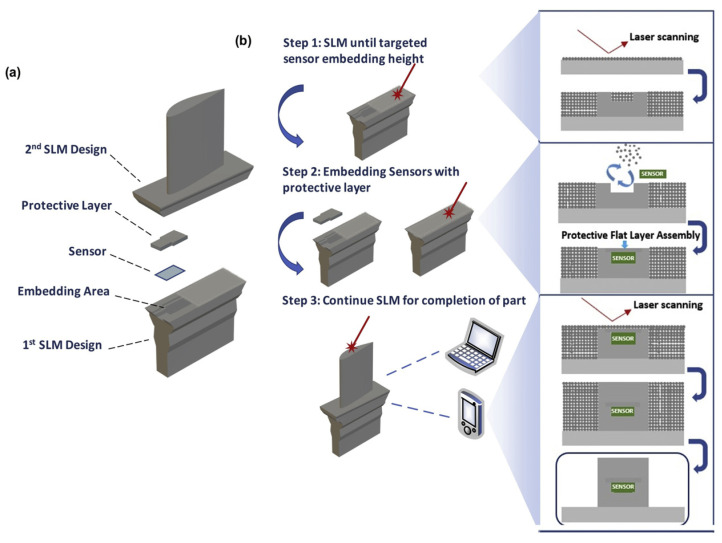
Schematic of sensor embedding selective laser melting (SE-SLM). (**a**) Design configuration of parts for the intermittent SLM process. (**b**) Three primary steps and details of the SE-SLM process [57]. Copyright 2020, Elsevier.

Jung et al. demonstrates an embedding method wherein integrated circuit chips are embedded into an Inconel 718C turbine in a SLM process to measure temperature and three-dimension vibration [57]. The turbine is printed in three steps: one which leads up to the embedding area, where the IC is embedded with the protective layer, a film. Next, the sensor is placed, and the third step consists of the SLM process continuing the part until it is finished, as displayed in Figure 13. Similar method called ‘stop and go’ has been demonstrated to integrate PZT sensors during EB-PBF AM technique by Terrazas et al. [106].

**Figure 14:**
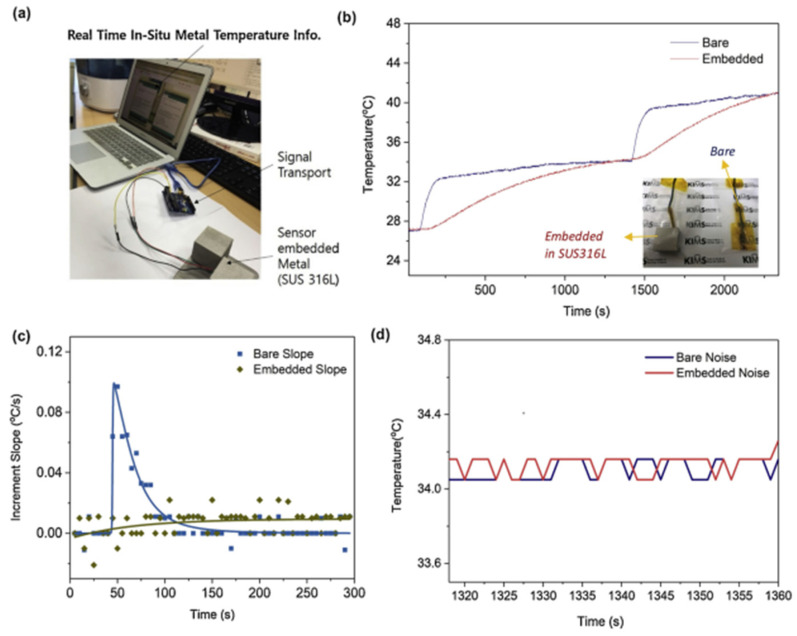
Validation of SE-SLM-processed temperature sensor operation by comparison with a bare temperature sensor. (**a**) Data reading set-up for monitoring the in-situ temperature of SE-SLM SUS316L. (**b**) Temperature profile comparison (**c**) Temperature increment slope profile (**d**) Noise level comparison [57]. Copyright 2020, Elsevier.

**Figure 15:**
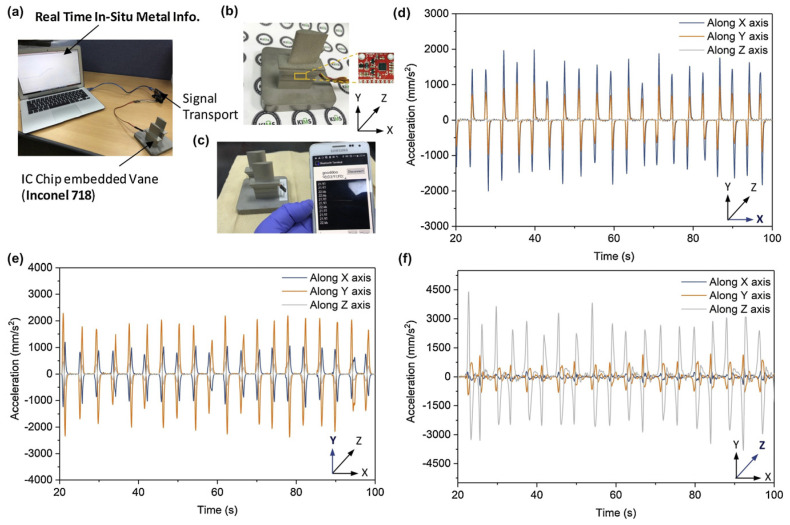
PCB-based IC component embedding in metal. (**a**) Setup for data reading from IC chip embedded in metal component. (**b**) IC chip-embedded Inconel 718 turbine vane. (**c**) Remote wireless monitoring of turbine vane temperature. (**d**–**f**) Recorded acceleration data for each axis vibration input ((**d**) X-axis, (**e**) Y-axis, and (**f**) Z-axis) [57]. Copyright 2020, Elsevier.

The performance of the embedded sensor was compared to a control sensor. The embedded sensor, despite having a different heating rate, reaches the same temperature as the bare sensor. This can be observed in each temperature elevation in Figure 14. The embedded and bare sensors also share the same amount of noise, around ±0.15 °C. These trends extend to the accuracy of the vibrational detection (Figure 15), although there is no control parameter of a bare sensor for comparison. Here, Jung et al. noted that the IC operates on Bluetooth, with a connection range of more than 100 m. 

### 3.5. DED Based 3D Printed Embedded Sensors

**Figure 16:**
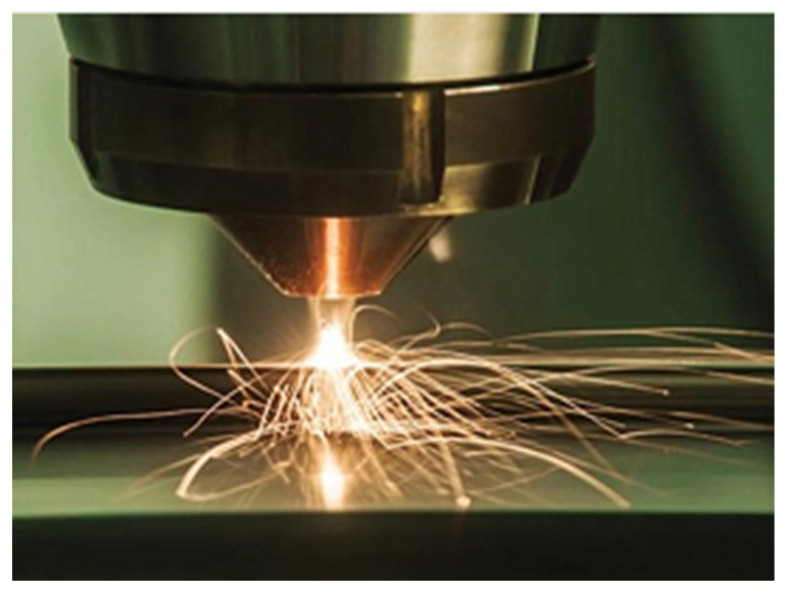
Image of laser powered DED process [107]. Copyright 2019, 3D natives.

Directed energy deposition (DED) is an AM process that uses a laser or electron beam to fuse material together as it is being deposited as shown in Figure 16 [107]. The material feedstock available for DED includes polymers, ceramics, and metals, but metals are the material that is mostly used in this approach and can be supplied as wires or powder [108]. One of the main issues that are prevalent when AM embedded sensors using DED is protection of the sensor from damage due to laser exposure and temperature damage. Juhasz et al. introduces a way to print embedded temperature-resistant strain sensors for metal dog bone specimens using DED [34]. The embedded sensors were printed into a thick sheet of high temperature resistant materials and multiple trials varied thickness of the sheet to monitor temperature degradation and damage to the sensor. The sensor sheets were placed during DED operation and in-situ interruption was programmed to embed the sensor into the dog-bone. To 3D print the dog bone specimens, a laser power of 375W, a mass flow rate of 3.23 g/min, a laser spot size of 1.7 mm and layer height of 1.02 mm were applied. The thickest 3D printed strain gauge sensor was the only to survive the DED process and had the ability to produce an output response when tensile stress was applied.

Kim et al. used DED to embed optical fiber sensors into a turbine blade for temperature scanning [109]. The significant elements that were investigated in this work was the optimization of printing parameters and implementing a material that is sensitive to detect the actual temperature. To prevent thermal damage to the sensor, they coated the fibers with Ni-alloy and implemented a print-and-stop procedure to allow heat to dissipate after each printed layer. The final wind turbine design was tested in extreme temperature conditions to test the detection ability of the sensors. Preliminary tests were done prior to ensure there were no defects during printing. 

### 3.6. Inkjet Based 3D Printed Embedded Sensors

**Figure 17:**
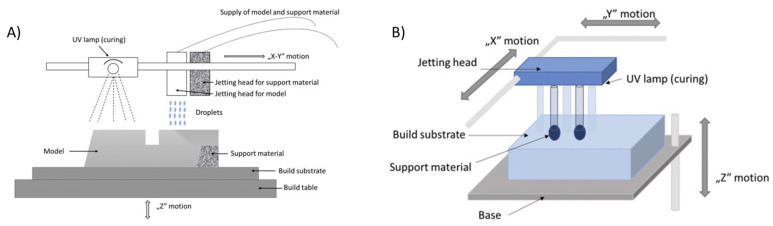
(**A**) MultiJet and (**B**) PolyJet printing schematics [110].

Inkjet 3D printers work by utilizing piezoelectric inkjet technology in order to release droplets of material on a bed. Each material layer deposited is cured before the following layer. Inkjet printing can be operated with two different methods: drop-on-demand (DoD) and continuous inkjet printing (CIJ) [110]. Based on this technology, two multi-material technologies were created: PolyJet (Stratasys Objet Geometries Co) (Figure 17A) and MultiJet (3D Systems) (Figure 17B). PolyJet and MultiJet use micronozzles that jet photopolymer resin droplets, liquid plastic material, or casting wax materials while simultaneously curing with UV light [111]. Gel-like support is used with both of these technologies. The key difference between these technologies is the print heads. MultiJet can have a maximum of two printheads. On the other hand, PolyJet can be comprised of two or more print heads.

Andò et al. successfully developed a flexible electromagnetic driven actuator using a low-cost Inkjet printer [112]. They additively manufactured a conductive coil onto a substrate made of polyethylene terephthalate (PET) and an external magnet. In addition, a strain gauge was 3D printed onto the PET beam which connects the magnet and coil to a patterned printed circuit board (PCB). Pinto et al. used PolyJet technology to manufacture stretchable conductors and pressure sensors. They aimed to create a rapid-manufacturing technique of microfluidic substrates embedded with liquid metals. In order to create the microchannels, the J750 (Stratasys) PolyJet printer and the Agilus30 (Stratasys) UV-cured resin were used. The 3D printing process was the following: first, fabricating the bottom substrate with channel cavities; second, filling the channel cavities with support liquid, and lastly, fabricate the top substrate directly on top of the bottom substrate. The flexible printed microfluidic substrate was then filled with EGaIn liquid metal [113]. Mieloszyk et al., using MultiJet printing, fabricates a polymeric structure with embedded fiber Bragg grating (FBG) sensor [114]. FBG sensors have many applications such as strain and temperature measurements, in addition to vibration-based methods. The material used was a rigid polymer that was manufactured into dog-bone structures in order to test the sensor. Based on these experiments, InkJet technology has proven to be a good additive manufacturing method for both flexible and rigid polymer-based embedded sensors that can be used in many applications.

## 4. Integrated Physical Sensors and Their Applications

### 4.1. Piezoelectric Sensor

**Figure 18:**
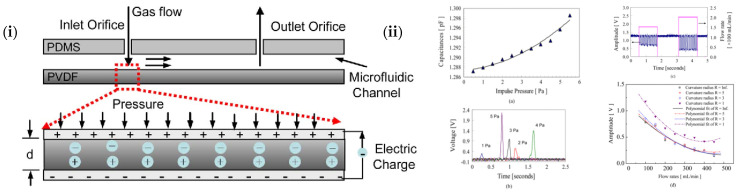
(**i**) The electric charge of the piezoelectric PVDF effect results from a deformation of the crystal lattice by changing the distance d when applying the pressure, producing a dipole moment. (**ii**) The results of PVDF microfluidic experimental data acquisition by LabVIEW software. (**a**) The capacitance values with the air flow impulses at differential pressures. (**b**) The voltages transfor-mation from capacitance by a charge amplifier. (**c**) The different frequency amplitudes with flow rates. (**d**) The output amplitude of the flow rates of frequency response versus the flow rate under different curvature radii [124], Copyright 2008, IEEE.

Piezoelectricity is a phenomenon that occurs in non-centrosymmetric crystals. When stress is applied to the material, it induces an electric polarization (charge). Conversely, when an electric field is applied, it induces a strain that is proportional to the field strength which is known as the converse effect and is used for actuation. The direct effect, on the other hand, is used for sensing changes in dynamic pressure, acceleration (from vibration or shock), and force [115]. Piezoelectric sensors are extensively utilized in various fields, including biomedical applications, ultrasonic imaging [116], energy harvesting [117,118,119], sensors [80], military and marine applications [120], automobile industry, and electronic devices [121] due to their remarkable mechanical, piezoelectric, and acoustic properties, making them ideal for everyday applications. Piezoelectric materials offer a new alternative for rapidly developing advanced electronic devices to replace traditional materials. As an example, in a study conducted by Zeyu et al., a 3D printed BTO-based piezoelectric ultrasonic transducer was developed which was able to focus energy and sense ultrasonic waves up to 6.28 MHz. The team successfully visualized the structure of a porcine eyeball using this transducer [122]. Tariverdian et al. conducted a study where they created 3D-printed scaffolds made of a composite material comprising barium strontium titanate (BST) and β-tricalcium phosphate (β-TCP) with interconnected macropores. These implantable materials were further analyzed to determine their ability to promote bioactivity and piezoelectricity, which are essential for bone healing [123]. Wen-Yang et al. developed a flexible piezoelectric pressure sensor for microfluidic applications. The sensor was made of PVDF sheets and PDMS and used microelectromechanical systems (MEMS) technology to create sensing patterns on the PVDF sheets. A molding transfer was designed to form the microfluidic channels of the PDMS, which were then integrated together (Figure 18). The piezoelectric microfluidic sensor could measure impulse pressure and flow rates resulting from electric charges generated when the sensor was mechanically deformed [124].

### 4.2. Piezoresistive Sensor

**Figure 19:**
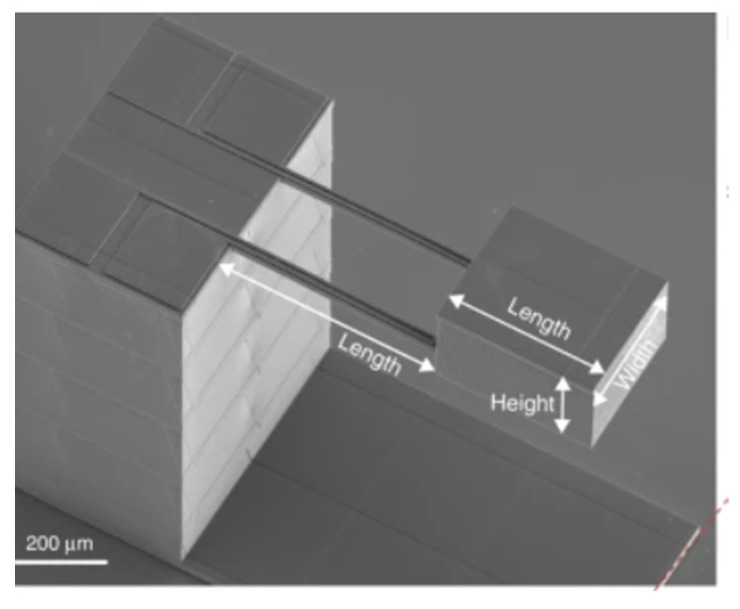
SEM image of 3D printed accelerometer by Pagliano et al. [126], Copyright 2022, Springer Nature.

The working principle of piezoresistive sensors is that of the change of the material’s electrical resistance caused by the application of mechanical stress. Piezoresistive sensors have been mentioned to be the sensors that are most vastly used on micro-scale and macro-scale devices [125] The materials mostly used for these types of sensors are semiconductors such as silicon, germanium, and polymers, which exhibit piezoresistive characteristics. These materials are mostly seen in microelectromechanical system (MEMs) devices (i.e., pressure sensors, microfluidic devices, accelerometers), where the substrate is often a rigid silicon that can be small and precise. Pagliano et al. successfully additively manufactured a functional MEMs accelerometer using two-photon polymerization with metal evaporation that is shown in Figure 19. This accelerometer successfully resembled the working principle of a piezoresistive sensor, therefore, the bending of the 3D printed cantilevers leads to the strain of the metal strain gauges and to the expected change of the electrical resistance of the strain gauges [126].

**Figure 20:**
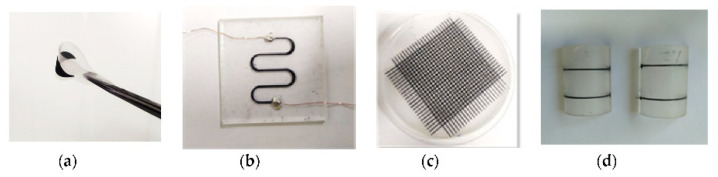
3D printed MWCNT-PDMS material patterns on soft substrates: (**a**) 3D printed film with extreme flexibility and bendability which shows that the sensor can be attached to non-conformal surfaces in practical applications; (**b**) 3D printed stretchable serpentine shape; (**c**) 3D printed grid forming 576 “taxels”; (**d**) printed MWCNT-PDMS composite on a non-conformal surface by Fekiri et al. [131], Copyright 2020, MDPI.

Recently, alternative materials, mostly composites such as carbon-based inclusions and metal nanoparticles infusions, have been developed for this application. These new composite materials allow for the fabrication of flexible piezoresistive sensors. The ability to additively fabricate flexible piezoresistive sensors has proven to be beneficial in different applications. Some applications include embedded pressure sensors in tires [127], wearable electronics [82], airflow sensors [128], food monitoring [129], and pneumatic actuators [130] among many others. In 2020, Fekiri et al. 3D printed flexible piezoresistive pressure sensors using a composite fabricated with a dispersion of multi-walled carbon nanotubes in polydimethylsiloxane (MWCNT-PDMS composite) via direct ink write (DIW) AM process [131] represented in Figure 20. They showed the feasibility of attaching their 3D printed sensors to non-conformal surfaces in addition to its flexibility and bendability [126]. The applications of piezoresistive sensors mentioned successfully show the many attributions that these sensors offer to advancement in different technologies. Piezoresistive sensors provide a means of converting mechanical changes into electrical resistance changes creating electrical signals, all in cost-efficient, compact parts.

### 4.3. Magnetic Sensor

The main function of magnetic sensors is to detect the strength, prescence, or direction of magnetic fields. The most common technique regarding additive manufacturing and embedding of magnetic sensors is by using Hall effect sensors, which generate a difference in voltage when exposed to magnetic field parallel to current flow [132]. Sensing devices are invaluable to the medical industry. Whenever there is an opportunity to perform invasive surgery, it is usually beneficial to do so. Chatzipirpirdis et al. fabricated a catheter tip for minimally invasive surgery, replete with a magnetic sensing tip [133]. Chatzipiripiridis et al. utilized 3D printing to develop a sensor base, which the magnetic sensor was embedded into, all encapsulated with a biocompatible PDMS tube. The magnetic sensor was calibrated to output newtons, all in an effort to either log forces undertaken by tissue or to characterize tissue for diagnosis. The development of the integrated sensor was successful, demonstrated by the sensing of the resistant force by raw beef. 

Olivas et al. also utilized Hall effect sensors in the creation of their 3D printed magnetic flux sensor system [134]. This approach combined additive manufacturing and micro dispensing, leading to very fine details in conductive traces. The researchers detail iterations of the magnetic flux sensing system, elucidating the decrease in package size. This work results in placement of electronics on curved surfaces, three-dimensional sensing, and surface-mount packaged electronic devices.

Using an alternative approach, Zhang et al. demonstrated the capability of magnetic hall sensors by fabricated a polymeric magnetic sensor based on a Mach-Zahnder interferometer [53]. The initial step in the creation of this sensor entailed the 3D printing of the device- a structure with a hollow cavity and two open channels that connect. After printing, the channels are infiltrated with magnetic fluids and sealed. When the magnetic field of the magnetic fluid varies, the refractive index of the fluid also changes, allowing an opportunity for detection. Ultimately, the researcher’s data supports sensing capabilities finer than Hall effect sensors in the nT range with anti-electromagnetic interference capabilities. 

### 4.4. Capacitive Sensor

The utilization of capacitive sensors within the realm of 3D printing presents a promising avenue for integrated sensor technology. A variety of applications have emerged that harness the potential of 3D printed capacitive sensors. For instance, in 2013, Shemelya and colleagues engineered touch capacitive sensors using Fused Deposition Modeling (FDM) technology, demonstrating the capability not only to detect touch but also to differentiate between various materials. These sensors find practical use in diverse domains, including biomedical sensing, human-machine interfaces, material analysis, electronics characterization, and environmental monitoring [27]. Moreover, researchers Lokesh Saharan and Toluwalase Agbesoyin ventured into 3D printing to develop capacitive sensors tailored for biomedical applications [135]. Gianni Stano and his team introduced an innovative method for single-step Additive Manufacturing, creating cost-effective capacitive sensors designed for liquid level measurement. Their work underscored the potential of FDM technology, achieving high sensor performance at an astonishingly low manufacturing cost of 0.38 € [136]. In addition, Chao Zhang and associates explored Digital Light Processing (DLP) 3D printing to craft versatile building blocks that could be configured into 3D flexible tactile sensors. These sensors included gyroid-based piezoresistive and gap-based capacitive sensors, exemplifying the adaptability of 3D printing in sensor creation [137]. These applications collectively highlight the diverse and transformative potential of 3D printed capacitive sensors across various sectors.

### 4.5. Gas Sensor

Gas sensors have gained attention due to the health concern posed by hazardous gases in society. Therefore, fabricating a gas sensor that is sensitive to a specific gas is essential. Applications for gas sensing include national defense, chemical process control to industrial manufacturing, and indoor/outdoor air quality control [138]. Gas sensing materials such as metal oxides have been extensively studied and the mechanism is well defined. The primary sensing mechanism involves gas adsorption induced charge transfer [55] and doping [105]. Commercial metal oxide sensors require high temperature for optimum selectivity and sensitivity performance. Therefore, integrated joule heating elements are used to reach high temperatures. In AM, Khan and Briand manufactured a fully printed metal-oxide gas sensor on a polyimide substrate by using aerosol jet and inkjet technologies. The all-printed metal-oxide gas sensor was able to obtain acceptable chemo-resistive response for CO and NO_2_ (Reducing, and oxidizing) compared to conventional metal-oxide gas sensor response. This work demonstrates future application of metal oxide gas sensors in portable smart printed electronics, and disposable systems [139].

### 4.6. Particle Sensor

**Figure 21:**
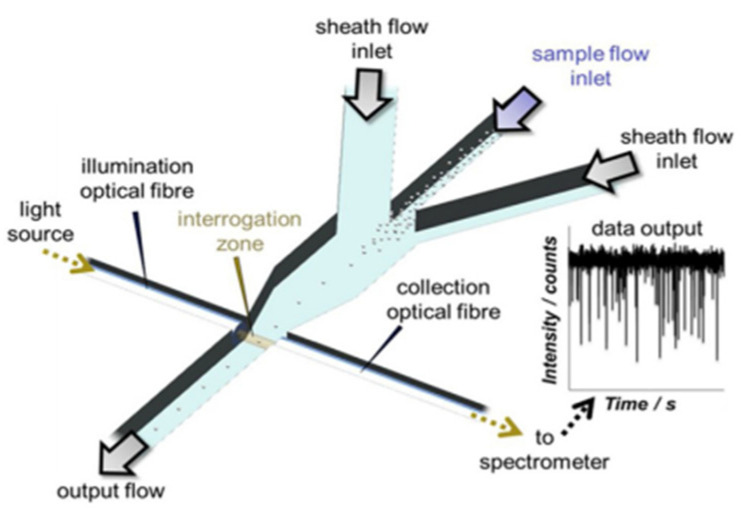
Schematic of the 3D printed microfluidic device [59]. Copyright 2017, Elsevier.

**Figure 22:**
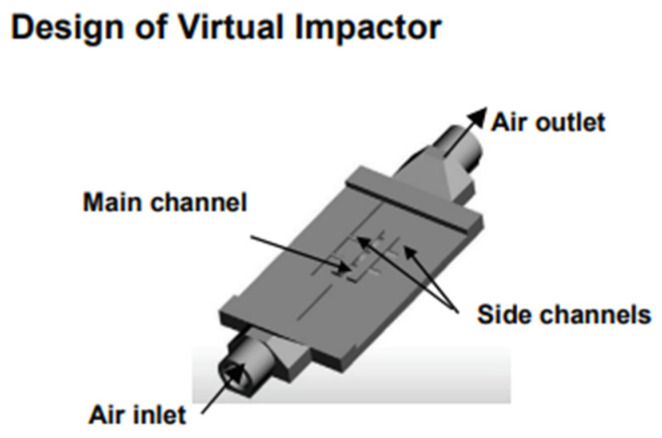
Schematic of the microchannels that form the virtual impactor in the miniature sensor [141]. Copyright 2018, AMA.

Particle sensors have the ability to detect particulate matter in the atmosphere and can be considered as tool for assessing pollutants [140]. This type of sensors can be successfully fabricated with AM by incorporating channels. A variety of applications can come to be by incorporating sensors to 3D printed channels, such as collection and detection of particles, health diagnostics, pharmaceutical manufacture, and environmental applications. Microfluidic devices have been researched and fabricated recently for particle sensing [59] shown in Figure 21. These microfluidic devices can be used for cell counting and synthesis applications. Hampson et al. successfully manufactured a microfluidic particle counter with stereolithography (SLA) technology with three different build directions [46]. This particle sensor was able to count particles up to a certain size and the different sizes that could be found in a mixture. It has been shown that successful particle detection can also be achieved with these sensors. Wang et al. 3D printed a miniature sensor with microchannels (as displayed in Figure 22) that function as virtual impactor and sort airborne particles by size and mass [141]. They used digital light projection (DLP) technology for this research. The particle detection happens by capacitive sensors. This miniature sensor proved to be a system that can be used in daily personal health monitoring.

### 4.7. Tactile Sensor

**Figure 23:**
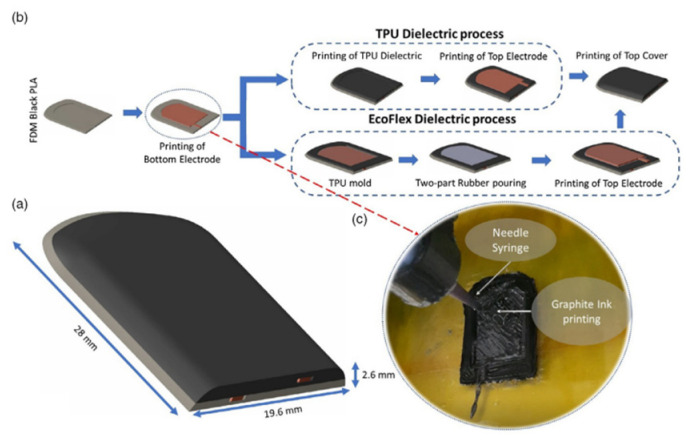
Tactile sensor phalanx structure of robotic hand; (**a**) whole phalanx structure, (**b**) fabrication procedure, (**c**) 3D printing via cold extrusion [143]. Copyright 2019, Wiley Online Library.

Tactile sensors are used to measure force or receive contact information such as strain, pressure, humidity, sound, and temperature, by outputting an electrical signal upon excitation [109]. Tactile sensors have four types of working mechanisms: piezoresistive, piezocapacitive, piezoelectric, and triboelectric [142]. The applications for this type of sensors range between intelligent systems (i.e., biometric devices and programs, automated systems), robotics and AI, object manipulation, human-computer interactions, healthcare, and biomedical engineering. With the benefits of AM, these sensors were able to be fabricated with soft and/or flexible materials in order to achieve higher commodity and feasibility of usage. Tactile sensors have been mostly used recently in soft robotic applications, mainly in robotic hands or grippers sensors [142,143,144,145]. James et al. successfully incorporated a commercial 3D printed tactile sensor to a three-dimensional-printed, three-fingered tactile robot hand. It was demonstrated that this robotic hand was able to distinguish and classify objects just by using tactile information that is stored and processed through a neural network. Ntagios et al. 3D printed their own tactile sensors which were later embedded on a 3D printed hand [143]. These tactile sensors where tested and proved that they can detect pressures as low as 1 kPa. Visual expression of these sensors are in Figure 23. Michaelis et al. have shown highly reproducible, hysteresis-free, flexible strain sensor fabrication by inkjet printing technology [24].

**Figure 24:**
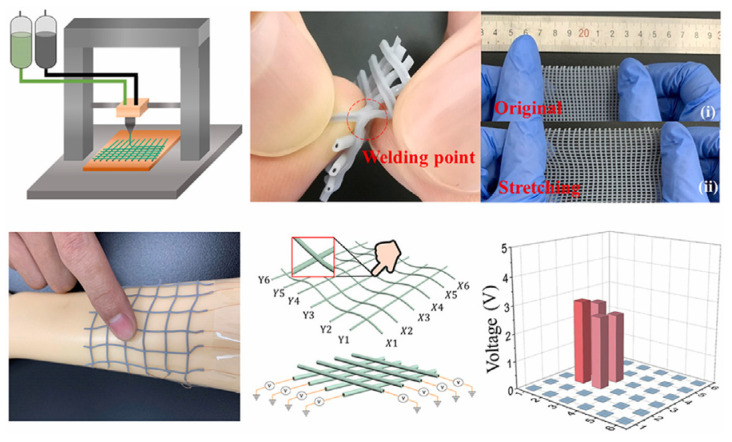
3D printed e-skin with incorporated tactile sensor [47]. Copyright 2021, Elsevier.

By fabricating flexible tactile sensors, the use of these structures has grown in healthcare and biomedical applications [61,146]. Chen et al. 3D printed flexible smart fibers and textiles to serve as e-skin. E-textiles have been previously applied to prostheses; however, e-skin is a new technology that strives to combine sensor and human skin directly shown in Figure 24 [47]. This e-skin was manufactured via DIW with the use of two mixtures: PDMS and graphene and PDMS and PTFE. A triboelectric effect was used to achieve the function of a tactile sensor.

### 4.8. Biosensors

**Figure 25:**
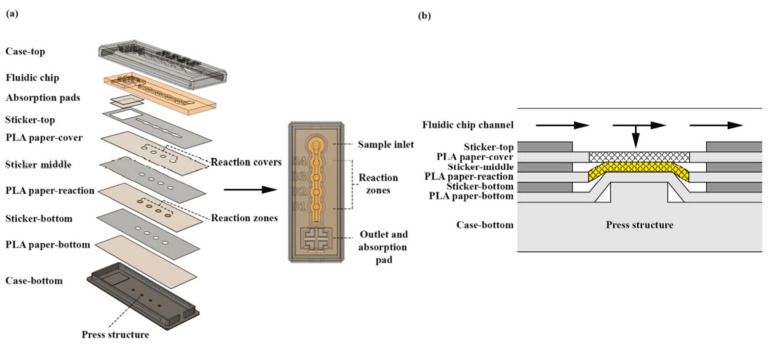
Illustration of the dengue virus serotype biosensor prototype assembly and components. (**a**) The prototype consists of the Fluidic chip component. (**b**) The cross-section illustrates the press structure that pushes the reaction zone (yellow mesh area) up against the reaction cover (white mesh area) [148]. Copyright 2021, Elsevier.

**Figure 26:**
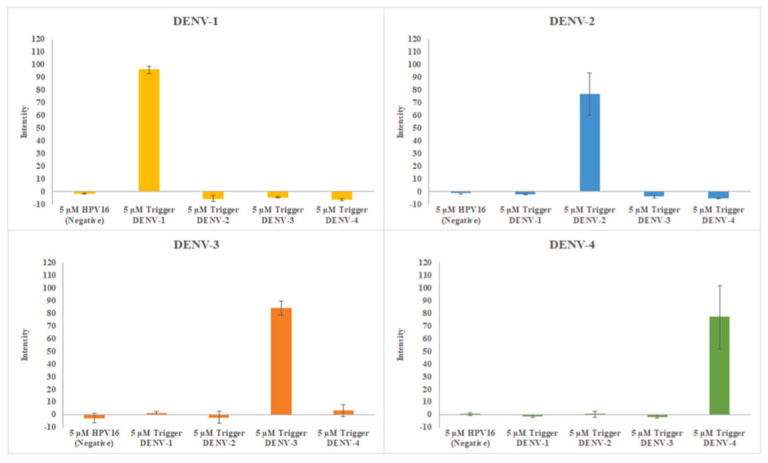
Specificity of RNA toehold switches for dengue virus serotype detection in cell-free reactions. The bar graphs were generated from the mean and ±SD (n = 3) of the color intensity from the cell-free reactions containing the RNA toehold switches (DENV-1 to DENV-4) that were exposed to 5 μM Trigger-DENV-1 to Trigger DENV-4 and HPV16 (negative control) [148]. Copyright 2021, Elsevier.

Biosensors are devices designed to measure biological reactions by generating a proportional signals to the concentration of the analyte [147]. The “analyte” is the substance that is to be detected. Many components are included within a biosensor, such as a bioreceptor, which is a molecule that recognizes the analyte. A transducer is needed to convert this bioreaction to an electrical reaction shown on an electrical display. AM provides a host of benefits to this phenomenon, such as freedom of design, rapid manufacturing for point-of-care testing, and fine features to a micro level. Pregnancy tests and recent rapid COVID-19 tests are prominent examples of biosensors. Suvanasuthi et al. demonstrated a 3D printed biosensing prototype that detects and discerns dengue virus serotypes [148]. Dengue is a mosquito-borne disease most prevalent in sub-tropical environments, with symptoms ranging from mild to severe. The need to detect various Dengue virus types is great, as DENV− 2 or DENV− 3 increases the chances of life-threatening disease. The researchers have printed a sensor integrated in a structure with two types of printing methods- material extrusion and vat photopolymerization. Material extrusion was used to print PLA and wax microfluidic paper-based analytical devices, where vat photopolymerization was utilized to fabricate the fluidic chip. RNA toehold switches served as the inspiration for the detection reaction for these sensors, where the switch would bind sequences of each dengue virus serotype. These triggers were embedded in the 3D printed papers. The fluidic chip helps prevent the sample from flowing to the absorption pads, giving enough time for the reaction. This housing is concise, as seen in Figure 25. The sensors proved to be very specific regarding their ability to discern between the serotypes, as shown in Figure 26. 

### 4.9. Chemical Sensor

Chemical sensors are very similar to biosensors, but rather than detecting biological information, they detect chemical information. Like biosensors, they also rely on the interaction of the analyte and receptor. This reaction is transduced to an electrical signal and then interpreted by the user. Common uses of chemical sensors include household carbon monoxide sensors and breathalyzers [149].

**Figure 27:**
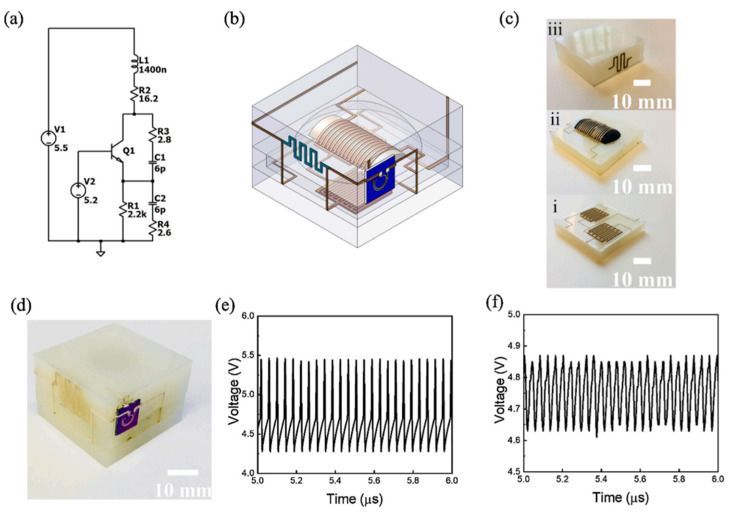
Characterization of a 3D integrated electrical oscillator. (**a**) Equivalent circuit of Colpitts oscillator. (**b**) Schematic of the 3D integrated electrical oscillator. (**c**) Fabrication procedure for the embedded oscillator. (**d**) Image of the printed 3D oscillator. (**e**) LTspice simulation of AC signal produced by Colpitts circuits. (**f**) Experimental output from the 3D-shaped oscillator [150]. Copyright 2021, Elsevier.

**Figure 28:**
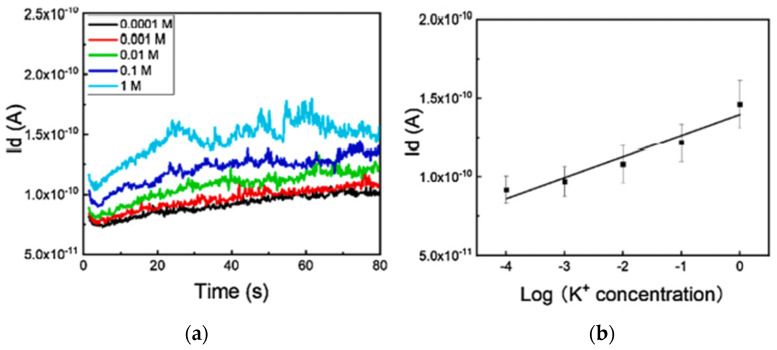
(**a**) Performance of the 3D integrated neuromorphic system for sensing various K+ ion concentrations. (**b**) Normalization of output from the fabricated 3D integrated neuromorphic sytem [150]. Copyright 2021, Elsevier.

Bao et al. developed a 3D-printed integrated neuromorphic sensor that mimics sensing in an organism. All components were 3D printed and assembled, including the sensor, oscillator, and transistor. This system can detect ion concentrations and was applied to discern low nutrient concentrations in soils. This complex system required the use of multiple printing processes, from material extrusion for the substrates to direct ink write for the inductors, capacitors, and resistors. The integrated system, seen in Figure 27, is assembled in three different layers, starting with the bottom capacitors, then the inductors, and finally the resistors [150]. The system began to monitor K^+^ ion concentrations in soil, as seen in Figure 28.

## 5. Challenges and Future Prospects

AM of integrated sensors offers many benefits including customization, cost savings, and faster production. Nevertheless, there are several challenges within 3D printing integrated sensors. Ensuring accurate and repeatable sensing performance from 3D printed sensors is difficult and needs careful control of the printing process and material selection. Selection of the right material for 3D printing that has the necessary electrical and mechanical properties for sensors can be challenging. A 3D printed sensor’s sensitivity and response time could not be as high as a conventional sensor’s, which could restrict their applications in some areas. Over time, 3D-printed sensors can experience performance deterioration due to issues such as material aging or exposure to harsh environments.

The future of 3D-printed integrated sensors is bright despite these difficulties. New materials are being created that offer better performance and stability as technology advances. The accuracy and dependability of 3D-printed integrated sensors are projected to increase as 3D printing technology develops, increasing their utility in a range of applications. 3D printing can minimize the cost of producing sensors by eliminating the requirement for specialized tooling and machinery. New sensors and products may launch faster due to 3D printing’s quicker production rate. The possible uses of 3D printed integrated sensors are further increased by the customization abilities of 3D printing, which enable the development of sensors with special and distinctive functionality. Integrated sensors are the increasing trend towards the use of artificial intelligence (AI) and machine learning (ML) techniques to improve sensor performance. AI and ML algorithms can analyze large amounts of data collected by integrated sensors and identify patterns and trends that might not be immediately apparent to human observers. This can help to improve the accuracy and reliability of sensor readings, as well as identify potential issues or problems before they become more serious. Additionally, an important development in the field of integrated sensors is the growing use of wireless communication technologies. Many integrated sensors are now capable of transmitting data wirelessly, allowing for real-time monitoring and analysis. This can be particularly useful in applications such as environmental monitoring or industrial automation, where it may not be practical to physically connect sensors to a centralized data collection system. Overall, despite having few challenges, 3D-printed integrated sensors have promising futures and can revolutionize the sensor industry.

## 6. Conclusions

This review discussed 3D-printed integrated sensors on structures during AM technology. Various materials and their 3D printing methods during the integration of sensors have been broadly analyzed. Different 3D printing methods for sensor integration and the application fields have been reviewed in this article. Future 3D printing with integrated sensors has a wide range of exciting potential. Targeted medicines can be delivered using 3D-printed sensors in medical implants and gadgets that monitor vital signs and follow the healing process. By adding 3D printed sensors to robots and AI systems, their sensory capacities can be improved, making them more agile, intelligent, and responsive. Real-time data transmission and data collection are possible using 3D printed sensors in a range of Internet of Things (IoT) applications, including smart homes and industrial automation. 3D-printed sensors can be used to gather information on air quality, temperature, humidity, and other environmental variables in distant or challenging areas. Additionally, 3D-printed sensors can be incorporated into materials and constructions to track their performance and collect crucial information for design and optimization. These are only a few instances of the integrated sensor with 3D printing possibilities. Future applications for 3D-printed sensors are likely to be much more creative as technology progresses.

## Data Availability

No data is available or generated.
